# Neuroplastic Effects Induced by Hypercapnic Hypoxia in Rat Focal Ischemic Stroke Are Driven via BDNF and VEGF Signaling

**DOI:** 10.3390/ijms262412019

**Published:** 2025-12-13

**Authors:** Pavel P. Tregub, Pavel A. Chekulaev, Georgy M. Zembatov, Eugenia D. Namiot, Michael A. Ignatyuk, Dmitrii A. Atiakshin, Arseniy K. Berdnikov, Zaripat Sh. Manasova, Peter F. Litvitskiy, Vladimir P. Kulikov

**Affiliations:** 1 Brain Science Institute, Russian Center of Neurology and Neurosciences, 125367 Moscow, Russia; 2 Department of Pathophysiology, I.M. Sechenov First Moscow State Medical University, 119991 Moscow, Russia; 3 Scientific and Educational Resource Center “Innovative Technologies of Immunophenotyping, Digital Spatial Profiling and Ultrastructural Analysis”, RUDN University, 117198 Moscow, Russia; 4 Department of Ultrasound and Functional Diagnostics, Altay State Medical University, 656040 Barnaul, Russia; kulikov57@mail.ru

**Keywords:** hypercapnic hypoxia, ischemic stroke, neurorehabilitation, VEGF, BDNF, neuroplasticity

## Abstract

In this study, the neurorehabilitation potential of combined and isolated intermittent hypercapnia and hypoxia exposure was evaluated following photochemically induced cerebral thrombosis in rats. Particular attention was given to the roles of possible neuroplasticity mechanisms mediated by VEGF and BDNF, as well as the potential of hypercapnic–hypoxic interventions to synergistically amplify the therapeutic effects of pharmacological neuroprotectants during recovery. A total of 50 male Wistar rats were randomly assigned to five equal groups (*n* = 10 per group), each undergoing a course of respiratory interventions lasting 30 min per day for 15 sessions. The groups included (1) a normobaric hypoxia (PO_2_ ≈ 90 mmHg) group, (2) a permissive hypercapnia (PCO_2_ ≈ 50 mmHg) group, (3) a combined hypercapnic hypoxia (PO_2_ ≈ 90 mmHg, PCO_2_ ≈ 50 mmHg) group, (4) a control group, and (5) a sham-operated group. Following the rehabilitation protocol, animals exposed to hypercapnic hypoxia exhibited a two-fold reduction in stroke volume compared with controls, significant improvement in motor coordination (as assessed via the rotarod test), and marked upregulation of VEGF and BDNF expression within the ischemic brain region. Notably, only the HH group showed a decrease in serum neuron-specific enolase (NSE) levels. These findings indicate that hypercapnic hypoxia exerts a possible neurorehabilitative effect after focal ischemic injury, superior to that of isolated hypoxia or hypercapnia. Possible mechanisms underlying this outcome may involve activation of neurotrophic (BDNF) and angiogenic (VEGF) signaling pathways.

## 1. Introduction

The search for effective neurorehabilitation strategies following ischemic stroke remains one of the most pressing challenges in both clinical and experimental neuroscience. Hypoxic respiratory training is a promising therapeutic approach for neurological disorders, including ischemic stroke [1]. Several studies have indicated the therapeutic potential of intermittent hypoxic exposure in various experimental models of central neural system (CNS) injury, such as perinatal brain damage and cerebral ischemia [2,3,4]. There is also a recent growing body of research exploring the therapeutic efficacy of permissive hypercapnia. These studies indicate that controlled inhalation of carbon dioxide at safe concentrations may have neuroprotective effects in cases of hypoxic brain injury [5,6].

Hypercapnic hypoxia (HH) is a promising approach that combines normobaric hypoxia with permissive hypercapnia [7]. Experimental evidence suggests that combined exposure to hypoxia and hypercapnia exerts a more pronounced neuroprotective effect than either intervention alone [8,9]. HH has demonstrated high efficacy as a stimulus for reparative and adaptive processes during the neurorehabilitation phase following ischemic stroke [10]. Regular sessions of hypercapnic hypoxia during the post-stroke recovery period have been shown to accelerate regenerative processes, improve motor function, and reduce neurological deficits. These effects have been observed both in vivo (in rat models) [7] and in clinical settings, where patients receiving HH treatment within 24–72 h after ischemic stroke exhibited improved recovery outcomes [10].

A wide range of mechanisms underlying the neuroprotective efficacy of combined hypercapnic and hypoxic exposure prior to experimental ischemic injury have been investigated [11]. Some signaling pathways—such as those involving HIF-1α and A1 adenosine receptors—are predominantly influenced by the hypoxic stimulus, while others—including those involved in NF-κB activation, antioxidant activity, inhibition of apoptosis, and maintenance of blood–brain barrier (BBB) integrity—are mainly modulated by hypercapnia [11]. Most of the molecular and cellular mechanisms contributing to the development of brain ischemic tolerance are mediated by the synergistic effects of both elevated CO_2_ and reduced oxygen availability; these include ATP-sensitive potassium channels, molecular chaperones, endoplasmic reticulum stress responses, and mitochondrial metabolic reprogramming [11].

Against this background, there is growing interest in exploring the neurorehabilitative potential of hypercapnic hypoxia (HH) as an independent therapeutic intervention aimed at modulating post-stroke neuroinflammatory, metabolic, and anti-apoptotic processes. It remains unclear how molecules that potentially drive better recovery following stroke—such as brain-derived neurotrophic factor (BDNF) and vascular endothelial growth factor (VEGF)—mediate neuroplasticity following HH exposure during recovery from cerebral ischemic injury. A question also arises regarding the existence of a connection between the potential neurorehabilitative effects of HH and the levels of BDNF and VEGF. Data on this connection could improve our understanding in this context.

This experimental study was conducted to investigate the mechanisms underlying the neuroplasticity induced by intermittent hypercapnic hypoxic exposure following ischemic brain injury to improve therapeutic outcomes during recovery. This study differs from previous in vivo research in that hypercapnic hypoxia is utilized not for ischemic preconditioning, but rather to explore its role in the rehabilitation of individuals after stroke.

## 2. Results

### 2.1. Motor Coordination and Neurological Deficit in Rats

The narrow beam walking test results (Table 1) demonstrated an overall improvement in motor coordination following the course of respiratory interventions in groups that included a hypoxic component. Specifically, a reduction in the slipping index was observed for both forelimbs and hindlimbs in the NbH group (by 30 and 25 p.p., respectively) and the HyperH group (by 20 and 28 p.p.). Despite these trends indicating a positive effect on sensorimotor function, the changes were not statistically significant when compared with the control group.

### 2.2. Rotarod Test Results and Neurological Deficit Assessment

In the rotarod test (Table 2), the NbH group exhibited a 48% reduction in latency to fall compared with pre-intervention values following completion of the rehabilitation course. No significant changes in this parameter were observed in the other groups before and after the respiratory intervention period.

However, the CSO group showed improved performance compared with the control group, with increases in latency to fall by 25 and 18 p.p., respectively.

Neurological deficit scores, assessed using the Neurological Severity Score (NSS) scale, did not show statistically significant differences either before and after rehabilitation or between experimental and control groups, with NSS values ranging between 2 and 4 points across all groups.

### 2.3. Infarct Volume in the Cerebral Cortex

**Figure 1:**
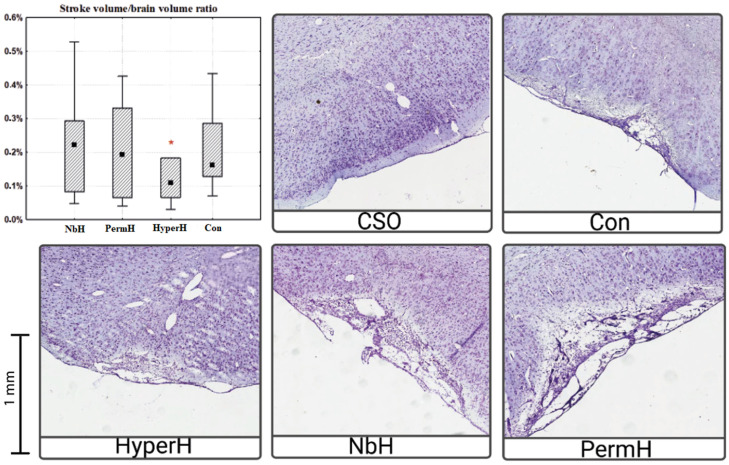
Stroke volume to brain volume ratio. * *p* < 0.05 compared with the control group. NbH—normobaric hypoxia; PermH—permissive hypercapnia; HyperH—hypercapnic hypoxia; Con—control group; CSO—control, sham-operated. The median value is indicated by the black square within the box.

The infarct volume, expressed as a percentage of total brain volume, was approximately two-fold lower in animals subjected to hypercapnic hypoxia compared with the control group (Figure 1). No statistically significant differences in infarct volume were observed between the other experimental groups and the control.

### 2.4. VEGF and BDNF Expression in Cells from the Ischemic Cortical Region

**Figure 2:**
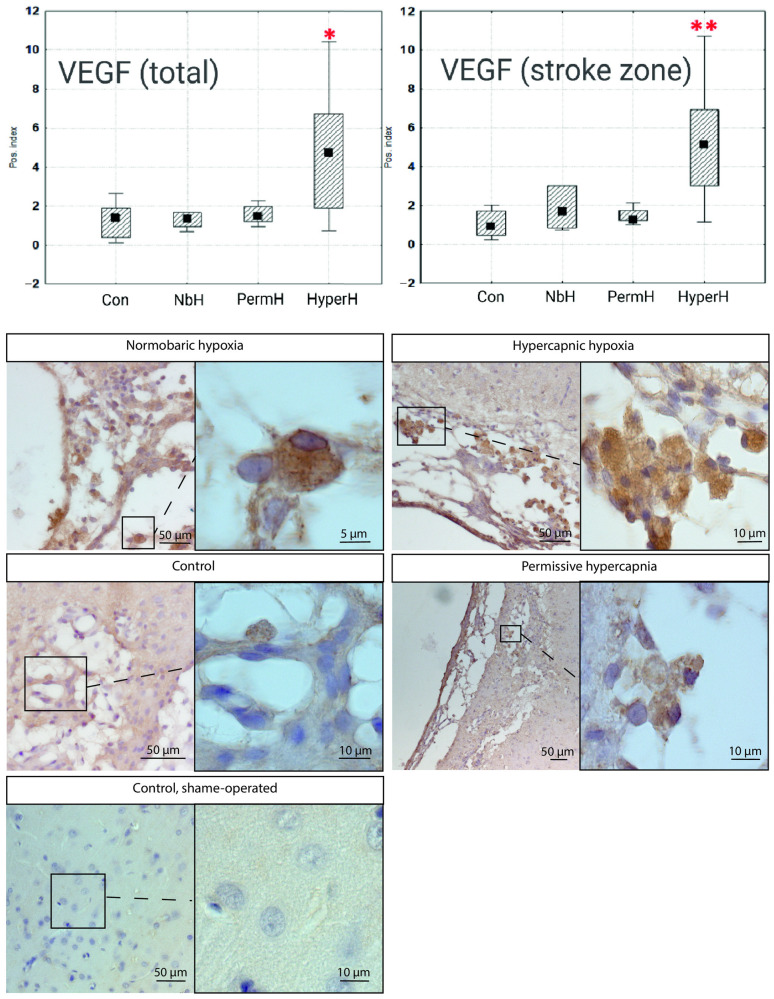
Ratio of VEGF-positive to VEGF-negative cells. * *p* < 0.05 vs. control; ** *p* < 0.001 vs. control. NbH—normobaric hypoxia; PermH—permissive hypercapnia; HyperH—hypercapnic hypoxia; Con—control group; The median value is indicated by the black square within the box.

The analysis of VEGF-positive cell indices revealed that only the number of VEGF-expressing cells in the HyperH group increased relative to that in the control group (Figure 2). In the ischemic lesion core, the VEGF positivity index was approximately five-fold higher in the HyperH group compared with the control.

No significant differences in VEGF expression in the peri-infarct area were observed between the experimental groups and the control group.

**Figure 3:**
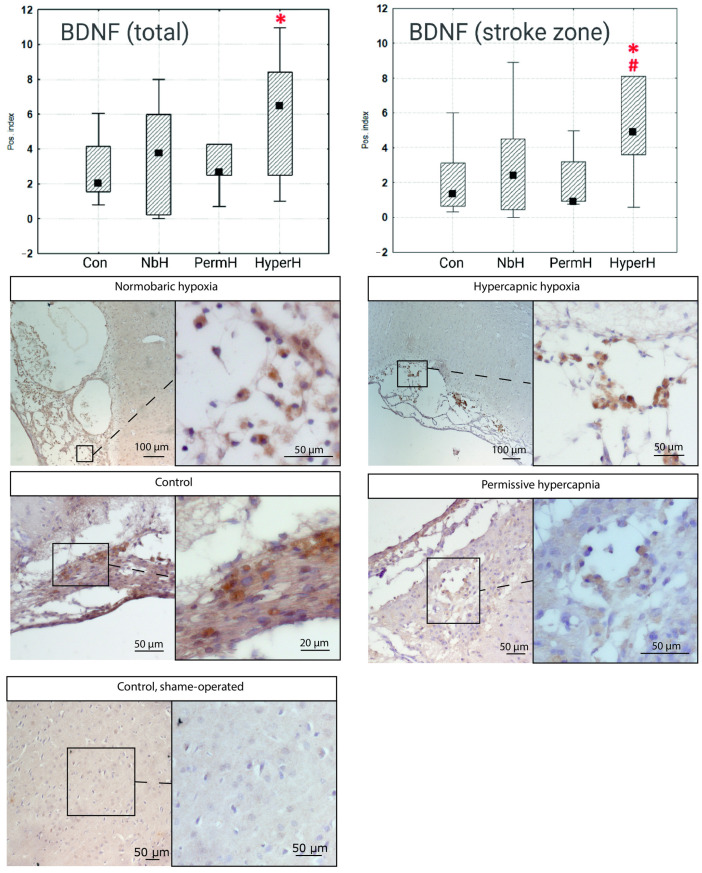
Ratio of BDNF-positive to BDNF-negative cells in the stroke core. * *p* < 0.05 vs. control; # *p* < 0.05 vs. NbH. NbH—normobaric hypoxia; PermH—permissive hypercapnia; HyperH—hypercapnic hypoxia; Con—control group; The median value is indicated by the black square within the box.

The BDNF-positive cell index measurements demonstrated a similar pattern (Figure 3). In the ischemic lesion core, the BDNF positivity index in the HyperH group differed significantly from those of the control and NbH groups. No significant differences between the experimental groups and the control were observed in the peri-infarct area.

### 2.5. Serum Levels of S100 and NSE in Rats

**Figure 4:**
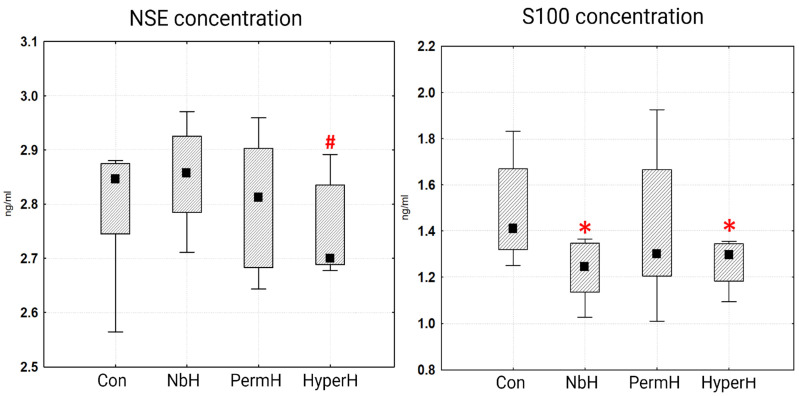
Serum levels of S100 and NSE. * *p* < 0.05 vs. control; # *p* < 0.05 vs. NbH; NbH—normobaric hypoxia; PermH—permissive hypercapnia; HyperH—hypercapnic hypoxia; Con—control group; The median value is indicated by the black square within the box.

Neural injury biomarker serum concentration measurements (Figure 4) revealed lower levels of S100 protein in the NbH (1.24 ng/mL) and HyperH (1.30 ng/mL) groups, compared with the control group (1.41 ng/mL).

The dynamics of NSE levels demonstrated a slightly different pattern. A significant reduction in NSE concentration was observed in the HyperH (2.70 ng/mL) group compared with NbH (2.86 ng/mL). No significant changes were observed in other groups.

## 3. Discussion

We have previously published several studies demonstrating the neuroprotective effects of hypoxia and hypercapnia as preconditioning strategies for inducing ischemic/hypoxic tolerance in the brain [7,8,9]. These studies consistently indicated that combined hypoxic and hypercapnic exposure was superior to either intervention alone. This enhanced efficacy is thought to involve multiple mechanisms, including HIF-1α upregulation, metabolic adaptations, apoptosis modulation, and preservation of blood–brain barrier (BBB) integrity [11]. Supporting these experimental findings, results from a randomized placebo-controlled clinical trial demonstrated the clinical efficacy of hypercapnic hypoxia as a neurorehabilitation strategy in the acute post-stroke period [10].

This study is the first to demonstrate the potential of intermittent hypercapnic hypoxia for neurorehabilitation—as opposed to neuroprotection—supported by histological and behavioral data. The functional roles of BDNF and VEGF in neurorehabilitation remain poorly understood, although previous preconditioning studies showed elevated VEGF serum levels after combined or isolated hypercapnic and hypoxic exposure [12].

In our earlier investigations, where hypoxia and hypercapnia were used as preconditioning tools to enhance cerebral ischemic tolerance, the combination of both stimuli consistently proved more effective than either hypoxia or hypercapnia alone [8,9]. However, when evaluating the rehabilitative potential of gas exposure protocols using the narrow beam walking test, no substantial differences were observed between the experimental groups. Notably, in the rotarod test, rats subjected to normobaric hypoxia alone showed worse performance following rehabilitation; this outcome may be attributable to compensatory hyperventilation-induced hypocapnia during the breathing sessions in this group—a phenomenon previously reported in the context of isolated hypoxic training [13]. Hypocapnia, which is a likely consequence of isolated hypoxia in the NbH group, fails to confer protection and instead induces neuronal damage, as evidenced by increased expression of the pro-apoptotic protein Bax, induction of mitochondrial damage, and recent clinical data [14,15].

In our study, no statistically significant differences in NSS were detected between the groups. This may be explained by the relatively small infarct size, which resulted in only mild sensorimotor deficits that could not be reliably differentiated between experimental conditions.

The Photothrombotic Stroke (PIT) model exhibits inherent variability in infarct volume due to individual differences in skull thickness and vascular anatomy [16]. While dye administration, laser parameters, and stereotaxic coordinates were standardized in our protocol to minimize this issue, some inter-animal variation persists. However, the key observation that only the HyperH group showed a nearly two-fold reduction in infarct size, while the NbH and PermH groups showed no significant difference from controls, indicates that the observed effects are treatment-specific rather than due to random variability.

Following intermittent hypercapnic hypoxia, we observed a reduction in infarct volume which was not observed in groups receiving isolated normobaric hypoxia or permissive hypercapnia. This supports the conclusion that the neurorehabilitative potential is maximized when both stimuli are combined. The mechanisms investigated within this study—specifically, those relating to BDNF and VEGF—may be involved in the neurorehabilitative potential of HH and, although a causal link between these mechanisms and post-stroke recovery under HH cannot yet be definitively established, these findings provide a basis for future investigation. It has been established that VEGF might play a critical role in post-stroke brain recovery by promoting both angiogenesis and neurogenesis [17]. However, the timing of VEGF activation is crucial: early VEGF delivery within the first hours after stroke may exacerbate blood–brain barrier (BBB) disruption, edema, and neuroinflammation, whereas administration several days post-injury has been associated with beneficial effects [18]. Similarly, upregulation of BDNF might positively contribute to recovery through stimulation of neuroplasticity and neurogenesis [19,20], further supporting the potential of hypercapnic hypoxia as a modulator of reparative processes in the post-stroke brain.

**Figure 5:**
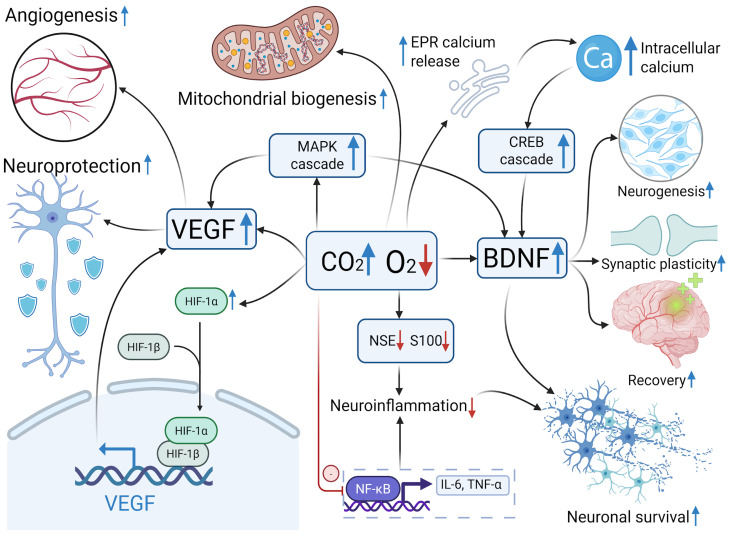
The roles of VEGF and BDNF in neuroplasticity under hypercapnic hypoxia. Blue arrows represent an increase or induction/activation, while red arrows reflect a decrease or inhibition. VEGF—vascular endothelial growth factor; MAPK—mitogen-activated protein kinase; BDNF—brain-derived neurotrophic factor; EPR—endoplasmic reticulum; CREB—cAMP response element-binding protein; HIF—hypoxia-inducible factor; NF-kB—nuclear factor kappa-light-chain-enhancer of activated B cells; TNF-α—tumor necrosis factor alpha.

One of the mechanisms potentially responsible for the increased expression of both VEGF and BDNF may involve activation of the MAPK pathway in response to hypercapnia [21,22]. The elevated VEGF expression observed in rats exposed to hypercapnic hypoxia may be linked to both the direct effects of hypoxia and hypercapnia on HIF-1α expression [23,24], as well as the indirect effect of MAPK activation triggered by hypercapnia. In contrast, BDNF expression may be regulated via a different, but complementary mechanism involving adenylyl cyclase activation and subsequent phosphorylation of CREB via PKA signaling [25]. Additionally, BDNF upregulation may result from hypercapnia-induced MAPK activity, which is known to activate the CREB/BDNF cascade [22,26]. It is also notable that MAPK activation can be induced by hypoxia alone [27]. Thus, the combined influence of hypoxia and hypercapnia on the MAPK signaling pathway may result in greater upregulation of BDNF and VEGF in neural cells compared with isolated exposures (Figure 5).

An important factor influencing the activity of secondary messengers and transcription factors is the intracellular calcium level [28]. Hypercapnia has been shown to increase the intracellular calcium concentration, primarily through its release from the endoplasmic reticulum [29,30], while some evidence suggests that extracellular calcium may also be involved [31]. Elevated calcium levels, in turn, activate CaMKII, leading to phosphorylation of CREB and enhanced BDNF expression [32].

Following exposure to hypercapnic hypoxia (including in combination with the JNK inhibitor), reductions in the serum levels of S100 and NSE were observed, whereas normobaric hypoxia resulted only in a decrease in S100. This difference may reflect more efficient restoration of neural tissue integrity and/or blood–brain barrier (BBB) function after hypercapnic hypoxia. These findings are consistent with our previous data demonstrating that combined exposure to hypercapnia and hypoxia led to the lowest degree of BBB permeability [11]. This BBB restoration effect is likely mediated by hypercapnia’s ability to suppress NF-κB activation, thus reducing the expression of pro-inflammatory cytokines (TNF-α, IL-6, IL-8) and adhesion molecules [33,34,35,36]. Furthermore, it directly inhibits TNF-α production [37] and pro-inflammatory interleukins while enhancing anti-inflammatory IL-10 [38,39]. Additional mechanisms include heat shock protein upregulation, further attenuating NF-κB responses [12,40]; metabolic reprogramming of macrophages toward the anti-inflammatory M2 phenotype [41]; and activation of mitophagy, which reduces inflammatory signaling [42]. Another factor that may contribute to restoring blood–brain barrier (BBB) integrity and suppressing neuroinflammation during hypercapnic hypoxia is the inhibition of matrix metalloproteinases (MMPs); in particular, it has been shown that hypoxic preconditioning can reduce the activities of MMP-2 and MMP-9—enzymes involved in increasing BBB permeability during ischemia–reperfusion injury [43,44].

It is important to acknowledge the limitations of this study. While the infarct volume was measured using serial sectioning, a technique that is both cost-effective and reliable, the inclusion of additional methods—such as TTC staining—could offer further insights into changes in infarct volume. In our study, we performed immunohistochemical analysis to investigate the roles of BDNF and VEGF in post-stroke recovery following gas exposure. Immunohistochemistry is a semi-quantitative method that provides preliminary data on BDNF and VEGF contents in the infarct region and on the correlations between these protein levels and clinical stroke outcomes. Utilizing quantitative methods—such as Western blot—could allow for more objective data quantification. Furthermore, to determine a causal relationship between the neurorehabilitative effect of hypercapnic hypoxia and BDNF/VEGF, the synthetic enhancement or inhibition of BDNF/VEGF is necessary. As this is the first study of its kind, the obtained data must be interpreted with caution. Further research using various methods, including by other research groups, is necessary to confirm the neurorehabilitative potential of hypercapnic hypoxia and to determine the presence of a causal link between BDNF/VEGF and improved post-stroke recovery.

Additionally, it is important to mention the potential risks associated with the clinical use of intermittent hypercapnic hypoxia exposures, which may include the development of cerebral tissue edema, increased systemic arterial pressure, and exacerbation of ischemia due to blood redistribution. The development of acidosis in neural tissue may also pose a potential danger.

## 4. Materials and Methods

### 4.1. Animals

The in vivo study was conducted using 50 male Wistar rats weighing an average of 250–300 g as animal models. A single animal was the experimental unit. The animals were previously kept in a specific antigen-free vivarium (SPF-vivarium) and were not sterilized, vaccinated, or subjected to other modifications, including genetic modifications. All experimental procedures were approved by the Local Ethics Committee of the Russian Center for Neurology and Neuroscience on 22 February 2021 under protocol code #81/2021, and were conducted in accordance with the principles of the European Convention for the Protection of Vertebrate Animals Used for Experimental and Other Scientific Purposes. Animals were randomized using Statistics for Windows 10.0 (StatSoft, Inc., Tulsa, OK, USA). Only the researchers involved in randomization and analysis of the results were aware of the group allocation at the different stages of the experiment. The rats were housed in standard cages under controlled room temperature (~23 °C) and natural light conditions, with ad libitum access to food and water. Body weights were recorded before and after the experimental procedures.

### 4.2. Groups and Experimental Design

**Figure 6:**
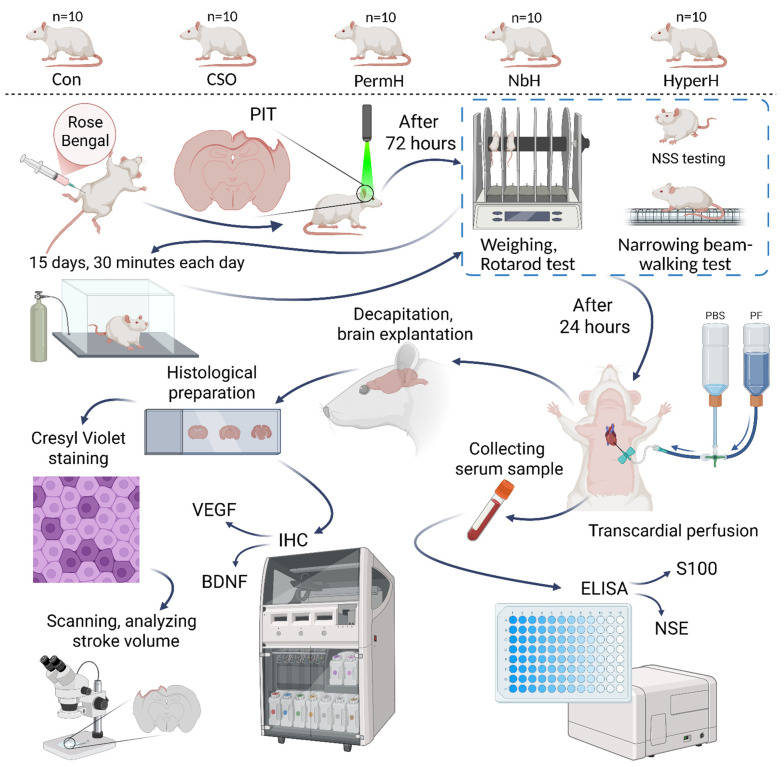
Experimental design. Rats were divided into equal groups. All animals, except those in the CSO group, underwent stroke modeling. Behavioral testing was performed 72 h post-stroke, followed by 15 days of respiratory training according to their group assignment. Upon completion of the respiratory training period, behavioral testing was conducted again. Subsequently, the rats were euthanized and tissue was harvested for further analysis (infarct volume measurement, ICH, ELISA). NbH—normobaric hypoxia; PermH—permissive hypercapnia; HyperH—hypercapnic hypoxia; Con—control group; CSO—control, sham-operated; PIT—photochemically induced thrombosis; BDNF—brain-derived neurotrophic factor; NSE—neuron-specific enolase; ELISA—enzyme-linked immunosorbent assay; VEGF—vascular endothelial growth factor; IHC—immunohistochemistry; PBS—phosphate-buffered saline; PF—paraformaldehyde.

The study consisted of a series of in vivo experiments and included five groups of animals (*n* = 10 in each group), which differed regarding the partial pressures of oxygen (PO_2_) and carbon dioxide (PCO_2_) during treatment (Figure 6).

The experimental design involved five groups of animals, each exposed to specific gas mixtures differing in partial pressures of oxygen (PO_2_) and carbon dioxide (PCO_2_):

- NbH (normobaric hypoxia): PO_2_ ≈ 90 mmHg, PCO_2_ ≈ 1 mmHg, and balance—nitrogen (N_2_).
- PermH (permissive hypercapnia): PO_2_ ≈ 150 mmHg, PCO_2_ ≈ 50 mmHg, and balance—N_2_.
- HyperH (hypercapnic hypoxia): PO_2_ ≈ 90 mmHg, PCO_2_ ≈ 50 mmHg, and balance—N_2_.
- Con (control group): PO_2_ ≈ 150 mmHg, PCO_2_ ≈ 1 mmHg, and balance—N_2_. Animals underwent all experimental procedures except gas exposure and breathed atmospheric air in the flow chamber.
- CSO (control, sham-operated): PO_2_ ≈ 150 mmHg, PCO_2_ ≈ 1 mmHg, and balance—N_2_. These animals underwent all procedures but received an injection of saline instead of Rose Bengal, thus not undergoing photochemically induced thrombosis (PIT); therefore, ischemia was not induced in this group. Animals breathed atmospheric air. The CSO group serves as an additional control of normal rat neural tissue during histological evaluation, immunohistochemical analysis, and behavioral testing.

We selected PO_2_ ≈ 90 mmHg to model moderate normobaric hypoxia (~12% O_2_ equivalent), which is sufficient to engage hypoxia-responsive pathways while avoiding severe hypoxemia and behavioral distress during 30 min sessions. PCO_2_ ≈ 50 mmHg represents permissive hypercapnia. The combined HH (PO_2_ ≈ 90 mmHg, PCO_2_ ≈ 50 mmHg) setting follows our prior work indicating the superior neuroprotective effects of HH in comparison with isolated hypoxia or hypercapnia, aligning with the rehabilitation intent of this study. Control atmospheres (PO_2_ ≈ 150 mmHg, PCO_2_ ≈ 1 mmHg) approximate room air in the chamber and serve as physiological baselines.

All animals (*n* = 50) were subjected to focal cerebral ischemia induced by the photothrombotic method [16].

At 72 h after ischemic injury, behavioral tests were performed, including the rotarod test, the narrow beam walking test, and the NSS assessment.

For the following 15 days, each group underwent daily 30 min exposures to their assigned gas mixture. Exposures were conducted in a sealed, custom-designed chamber ensuring stable and uniform distribution of gas partial pressures.

Upon completion of the respiratory training course, the animals were re-tested using the same behavioral paradigms, including the rotarod test.

At 24 h after the final behavioral assessment, animals were anesthetized and venous blood was collected from the inferior vena cava for ELISA-based evaluation of the neuroinflammatory markers S-100 and neuron-specific enolase (NSE). This was followed by transcardial perfusion fixation, decapitation, and brain extraction.

Subsequently, histological brain sections were prepared to determine stroke volume and conduct immunohistochemical analysis of BDNF and VEGF expression.

### 4.3. Assessment of Neurological Deficit and Behavioral Testing

#### 4.3.1. Neurological Severity Score

Before NSS testing [45], animals were monitored for signs of severe stress or abnormal motor activity unrelated to neurological deficits, as recommended in such protocols; however, no animals with these signs were detected in this study and none were excluded.

All tests were conducted under standard laboratory conditions (temperature 22–24 °C, moderate ambient lighting). Animals were acclimated to the testing room for 30 min prior to evaluation.

Neurological status was assessed using a 10-point scoring system, where 0 points indicated normal function and 10 points represented the most severe neurological deficit. The NSS test included the following parameters: locomotor ability, coordination (inclined plane test), reflexes (startle response, withdrawal reflex), movement symmetry, and exploratory behavior.

Each animal was evaluated for 5 min. The total NSS was calculated as the sum of points across all test items. Scoring was performed by two independent investigators, and the final result was the arithmetic mean (M) of their assessments. Testing was repeated on days 1, 3, and 7 after the experimental intervention to monitor the dynamics of functional recovery.

#### 4.3.2. Narrow Beam Walking Test

Sensorimotor function was assessed using the narrow beam walking test (OpenScience, Krasnogorsk, Russia) [46]. All trials were conducted under standard laboratory conditions (temperature 22–24 °C, moderate lighting). Prior to testing, animals were acclimated to the testing environment for 30 min.

The following parameters were recorded:

- Number of foot placements on the lower board (classified as errors);
- Number of slips (partial or complete paw displacement from the upper to lower board);
- Total number of steps from the starting point to the entrance of the dark compartment.

Individual outcomes were recorded for forelimbs and hindlimbs. Behavioral sessions were video-recorded and analyzed frame-by-frame using the RealTime software, version 7.6 (Minitab, LLC, State College, PA, USA). For each animal, data were averaged as the mean value (M) across two consecutive trials.

The degree of sensorimotor deficit was calculated using the formula:

Deficit (%) = [(Errors + 0.5 × Slips)/Total Steps] × 100

Testing was performed at three different time points to assess the dynamics of functional recovery. Animals were monitored for signs of severe stress or abnormal motor activity unrelated to neurological deficits. No animals showed such signs; thus, none were excluded. All evaluations were performed by two independent investigators, and mean values were calculated to minimize subjectivity.

#### 4.3.3. Rotarod Test

Motor coordination and endurance were assessed using the rotarod test [47]. All trials were performed under standard laboratory conditions (temperature 22–24 °C). Animals were acclimated for 30 min prior to testing and received habituation training on a stationary or slowly rotating rod (Rotarod for Mice and Rats, Model I-755, Campden Instruments, Loughborough, England, UK).

During testing, the rod rotation speed increased linearly from 4 to 40 rpm over 300 s (5 min), with a maximum test duration of 300 s.

Each rat was tested in three independent trials with 15–20 min rest intervals to avoid fatigue. The animal was placed on the rotating rod and their latency to fall (in seconds) was recorded, with 300 s as the maximum possible score.

If an animal fell within the first 10 s, the trial was repeated (no more than 3 repetitions per attempt). The mean latency (M) to fall across three successful trials was calculated for each animal. Additionally, the latency to the first fall and the number of repeated trials required were documented. Prior to the test, animals were monitored for signs of severe stress or abnormal motor activity unrelated to neurological deficits, as recommended in such protocols; however, no such animals were detected in this study and none were excluded.

To ensure objectivity, all assessments were performed by two independent observers and the results were averaged.

### 4.4. Respiratory Exposure Protocol

Respiratory interventions were performed using a custom-designed flow chamber previously described by Kulikov V.P. et al. [8]. Experimental rat groups were exposed to specific gas mixtures depending on their assigned treatment condition. Control and CSO animals were placed in the same chamber under identical conditions; however, atmospheric air was delivered (via a compressor) instead of a gas mixture. The composition of the gas atmosphere inside the chamber was continuously monitored using a Microlux O_2_+CO_2_ gas analyzer (Microlux Ltd., Chelyabinsk, Russia).

### 4.5. Surgical Procedure and Photochemically Induced Thrombosis

All animals were fasted overnight prior to the induction of focal cerebral ischemia, with unrestricted access to water. Anesthesia was induced via intraperitoneal injection of a solution containing tiletamine hydrochloride and zolazepam hydrochloride (25 mg/kg).

A sterile incision was made in the left inguinal region, and a 4% solution of Rose Bengal (Sigma Aldrich, Steinheim, Germany) in 0.9% NaCl was injected into the left femoral vein at a dose of 40 mg/kg.

Focal cortical ischemia was induced using the PIT method described by Barth and Mody [16]. A green laser (532 nm, 20 mW) was applied transcranially to the exposed skull for 10 min; in particular, a 2 mm diameter region on the right parietal bone was illuminated, located midway between the bregma and lambda sutures and 2 mm lateral to the sagittal suture, which photo-activated the dye and produced localized platelet thrombosis and arterial occlusion in the illuminated territory. Sham-operated animals received saline instead of dye under identical illumination to control for surgical/laser effects.

### 4.6. Histology and Measurement of Ischemic Lesion Volume

#### 4.6.1. Histological Preparation

Prior to decapitation, animals underwent transcardial perfusion fixation with 500 mL of phosphate-buffered saline (PBS), followed by 250 mL of 4% paraformaldehyde in PBS. Extracted brains were immediately post-fixed in 10% neutral buffered formalin for 24 h to preserve tissue morphology. After fixation, the brains were removed and the ischemic region was dissected using microsurgical tools (Monvet Ltd., Moscow, Russia).

The isolated fragment was placed into a histological cassette and processed in an automated tissue processor.

#### 4.6.2. Nissl Staining

Tissue samples were dehydrated in a graded ethanol series and embedded in paraffin. Paraffin blocks were sectioned at 10 μm thickness at 190 μm intervals throughout the infarcted region. Sections were stained with toluidine blue using the Nissl method and mounted in neutral plastic resin for digital scanning with a Leica Aperio CS2 microscope (Leica Biosystems, Deer Park, IL, USA). A subset of paraffin sections was reserved for immunohistochemical analysis.

#### 4.6.3. Infarct Volume Quantification

Sections were scanned, and the infarct area on each section was measured by a blinded investigator. Images were processed using the Aperio eSlide Manager 12.4.3 (Leica Biosystems, Deer Park, IL, USA). Infarct volume was calculated using the formula V = x × t × ΣS, where x is the interval between sections, t is the section thickness, and ΣS is the sum of infarcted areas across all sections [48].

### 4.7. Immunohistochemistry

Tissue sections were deparaffinized in xylene (3 × 10 min), followed by rehydration in isopropanol (3 × 5 min) and distilled water (5 min). Antigen retrieval was conducted in citrate buffer (pH 6.0) via heating in a water bath for 20 min at 95–98 °C, followed by cooling at room temperature for 20–30 min. Sections were then washed with PBS (2 × 5 min) and distilled water (5 min). Endogenous peroxidase activity was blocked with 3% hydrogen peroxide (15 min), followed by rinsing in distilled water (2 × 5 min).

Sections were incubated overnight at 4 °C with primary antibodies against BDNF (1:100, cat#PAA011Ra01, Cloud-Clone, Wuhan, China) and VEGFA (1:100, cat#PAA143Ra01, Cloud-Clone, Wuhan, China). After warming to room temperature, sections were rinsed with PBS (3 × 5 min) and incubated with HRP-conjugated secondary antibodies (cat#SAA544Rb59, Cloud-Clone, Wuhan, China) for 60 min at room temperature, followed by another PBS rinse (3 × 5 min). Signal detection was performed using a DAB substrate kit (Cloud-Clone, Wuhan, China), with the reaction visually monitored under a light microscope and halted by rinsing with distilled water. Dehydration was performed using isopropanol (3 × 3 min) and xylene (3 min, 4 min, 5 min).

Sections were then mounted under coverslips for digital scanning with a Leica Aperio CS2 microscope (Leica Biosystems, Deer Park, IL, USA). The number of immunopositive cells for BDNF and VEGF was counted together with the total number of cells in each section, and their percentage ratio (positivity index) was calculated. For each sample, the median positivity index was computed from the data of all sections belonging to that sample. Cell counting on each section was performed by a researcher blinded to the group assignments. Microphotographs were processed using the Aperio eSlide Manager 12.4.3 (Leica Biosystems, USA).

### 4.8. Enzyme-Linked Immunosorbent Assay (ELISA)

The ELISA was performed according to the manufacturer’s instructions (Cloud-Clone, China). All reagents were brought to room temperature (18–25 °C) prior to use. Standards were reconstituted in 0.5 mL of Standard Diluent to final stock concentrations of 10 ng/mL (S100) and 40 ng/mL (NSE), and then incubated for 10 min at room temperature with gentle mixing. A 7-point standard curve was prepared via serial two-fold dilutions in Standard Diluent, starting from 250 μL of the reconstituted standard. The final tube contained only Standard Diluent (0 ng/mL).

Reagents A and B were centrifuged and diluted to 1:100 using Assay Diluent A and B, respectively. The wash buffer was prepared by diluting 20 mL of concentrate in 580 mL of deionized water (final volume: 600 mL).

Each microplate well received 100 μL of either diluted serum sample or standard, and then was incubated for 1 h at 37 °C. Without washing, 100 μL of reagent A was added to each well and incubated for an additional hour at 37 °C. Wells were then washed three times with 350 μL of wash buffer (1–2 min per wash), followed by adding 100 μL of reagent B and incubation for 30 min at 37 °C. Wells were washed five times, and then incubated with 90 μL of TMB substrate for 10–20 min at 37 °C in the dark. The reaction was halted by adding 50 μL of Stop Solution, resulting in a color change to yellow.

The optical density was measured at 450 nm using a microplate reader (INNO-S, LTek, Seongnam-si, Republic of Korea). Background-corrected absorbance values (zero standard subtracted) were averaged across replicates. Standard curves were constructed using linear regression, plotting concentration (ng/mL) against absorbance.

### 4.9. Statistical Analysis

The sample size for the entire cohort and for each experimental group was calculated based on prior studies using a quantitative scale method [49]. Statistical analysis was performed using Statistics for Windows 10.0 (StatSoft, Tulsa, OK, USA). The Shapiro–Wilk test was used to assess the normality of data distributions, both within and between groups. In cases where the distribution was not normal, the significance of differences between groups was evaluated using the nonparametric Mann–Whitney U test, and that within a single group using the Wilcoxon test. Additionally, the Kruskal–Wallis one-way analysis of variance was performed to compare differences between groups. Quantitative data are presented as median (Me), lower quartile (25%), and upper quartile (75%). Differences were considered statistically significant at *p* < 0.05.

## 5. Conclusions

Normobaric hypoxia and hypercapnic hypoxia effectively reduced neurological deficits caused by focal ischemic injury. Among the experimental groups, the smallest infarct volume at the end of the rehabilitation course was observed in the group characterized by intermittent respiratory exposure to hypercapnic hypoxia. Furthermore, hypercapnic hypoxia led to increases in VEGF and BDNF expression within the stroke core, but not in the peri-infarct region. While these changes are noteworthy, they do not provide evidence of causation at this stage.

## Figures and Tables

**Table 1:** Narrow beam walking test results.

	Slipping Index							
	Forelimbs				Hindlimbs			
	After Stroke	After Treatment	Before and After Treatment	Compared with Control	After Stroke	After Treatment	Before and After Treatment	Compared with Control
Con	18.47	17.59	95%	-	23.21	25	108%	-
NbH	22.12	15.52	70% *	88%	30.77	23.21	75% *	93%
PermH	34.84	24.89	71%	142%	33.7	37.82	112%	151%
HyperH	15.56	12.5	80% *	71%	23.64	16.95	72% *	68%
CSO	19	17.65	93%	100%	24.97	23.28	93%	93%

Note: NbH—normobaric hypoxia; PermH—permissive hypercapnia; HyperH—hypercapnic hypoxia; Con—control group; CSO—control, sham-operated. * *p* < 0.05 compared with the control group. Color is used to highlight statistically significant results.

**Table 2:** Rotarod test results.

	After Stroke	After Treatment	Before and After Treatment	Compared with Control
Con	128.7	117.9	92%	-
NbH	151.15	78.625	52% *	67%
PermH	118.95	110.6	93%	94%
HyperH	138.65	142.3	103%	121%
CSO	137	138.6333	101%	118% *

Note: NbH—normobaric hypoxia; PermH—permissive hypercapnia; HyperH—hypercapnic hypoxia; Con—control group; CSO—control, sham-operated. * *p* < 0.05 compared with the control group. Color is used to highlight statistically significant results.

## Data Availability

Due to the large volume and file size of the primary data (high-resolution digital micrographs), the full dataset is stored on our institutional server and is not hosted in a public repository. However, all original files are fully available from the corresponding author upon reasonable request.

## Abbreviations

ATP	Adenosine triphosphate
BBB	Blood–brain barrier
BDNF	Brain-derived neurotrophic factor
CaMKII	Calcium/calmodulin-dependent protein kinase II
CREB	cAMP response element-binding protein
HIF	Hypoxia-inducible factor
PBS	Phosphate-Buffered Saline
PF	Paraformaldehyde
EPR	Endoplasmic reticulum
cAMP	Cyclic adenosine monophosphate
ICAM-1	Intercellular adhesion molecule 1
IL	Interleukin
JNK	c-Jun N-terminal kinase
MAPK	Mitogen-activated protein kinase
MMP	Matrix metalloproteinase
NF-κB	Nuclear factor kappa-light-chain-enhancer of activated B cells
NSE	Neuron-specific enolase
NSS	Neurological severity score
p.p.	Percentage points
PCO_2_	Partial pressure of carbon dioxide
PO_2_	Partial pressure of oxygen
TNF-α	Tumor necrosis factor alpha
VCAM-1	Vascular cell adhesion molecule 1

## References

[1] Yuan H., Liu J., Gu Y., Ji X., Nan G. (2022). Intermittent hypoxia conditioning as a potential prevention and treatment strategy for ischemic stroke: Current evidence and future directions. Front. Neurosci..

[2] Sharp F.R., Ran R., Lu A., Tang Y., Strauss K.I., Glass T., Ardizzone T., Bernaudin M. (2004). Hypoxic preconditioning protects against ischemic brain injury. NeuroRx.

[3] Pietrogrande G., Zalewska K., Zhao Z., Johnson S.J., Nilsson M., Walker F.R. (2019). Low Oxygen Post Conditioning as an Efficient Non-pharmacological Strategy to Promote Motor Function After Stroke. Transl. Stroke Res..

[4] Sprick J.D., Mallet R.T., Przyklenk K., Rickards C.A. (2019). Ischaemic and hypoxic conditioning: Potential for protection of vital organs. Exp. Physiol..

[5] Tao T., Liu Y., Zhang J., Xu Y., Li W., Zhao M. (2013). Therapeutic hypercapnia improves functional recovery and attenuates injury via antiapoptotic mechanisms in a rat focal cerebral ischemia/reperfusion model. Brain Res..

[6] Pruimboom L., Muskiet F.A.J. (2018). Intermittent living: The use of ancient challenges as a vaccine against the deleterious effects of modern life—A hypothesis. Med. Hypotheses.

[7] Kulikov V.P., Bespalov A.G., Yakushev N.N. (2008). The effectiveness of training with hypercapnic hypoxia in the rehabilitation of ischemic brain damage in the experiment. Bull. Restor. Med..

[8] Kulikov V.P., Tregub P.P., Bespalov A.G., Vvedenskiy A.J. (2013). Comparative efficacy of hypoxia, hypercapnia and hypercapnic hypoxia increases body resistance to acute hypoxia in rats. Patol. Fiziol. Eksp. Ter..

[9] Tregub P., Kulikov V., Motin Y., Bespalov A., Osipov I. (2015). Combined exposure to hypercapnia and hypoxia provides its maximum neuroprotective effect during focal ischemic injury in the brain. J. Stroke Cerebrovasc. Dis..

[10] Alekseeva T.M., Topuzova M.P., Kulikov V.P., Kovzelev P.D., Kosenko M.G., Tregub P.P. (2024). Hypercapnic hypoxia as a method of rehabilitation of patients after ischemic stroke. Neurol. Res..

[11] Tregub P.P., Kulikov V.P., Ibrahimli I., Tregub O.F., Volodkin A.V., Ignatyuk M.A., Kostin A.A., Atiakshin D.A. (2024). Molecular Mechanisms of Neuroprotection after the Intermittent Exposures of Hypercapnic Hypoxia. Int. J. Mol. Sci..

[12] Bespalov A.G., Tregub P.P., Kulikov V.P., Pijanzin A.I., Belousov A.A. (2014). The role of VEGF, HSP-70 and protein S-100B in the potentiation effect of the neuroprotective effect of hypercapnic hypoxia. Patol. Fiziol. Eksp. Ter..

[13] Varis N., Leinonen A., Parkkola K., Leino T.K. (2022). Hyperventilation and Hypoxia Hangover During Normobaric Hypoxia Training in Hawk Simulator. Front. Physiol..

[14] Lasso Pirot A., Fritz K.I., Ashraf Q.M., Mishra O.P., Delivoria-Papadopoulos M. (2007). Effects of severe hypocapnia on expression of bax and bcl-2 proteins, DNA fragmentation, and membrane peroxidation products in cerebral cortical mitochondria of newborn piglets. Neonatology.

[15] Scudellari A., Dudek P., Marino L., Badenes R., Bilotta F. (2023). Ventilation Targets for Patients Undergoing Mechanical Thrombectomy for Acute Ischemic Stroke: A Systematic Review. J. Clin. Med..

[16] Barth A.M., Mody I. (2011). Changes in hippocampal neuronal activity during and after unilateral selective hippocampal ischemia in vivo. J. Neurosci..

[17] Ceci C., Lacal P.M., Barbaccia M.L., Mercuri N.B., Graziani G., Ledonne A. (2024). The VEGFs/VEGFRs system in Alzheimer’s and Parkinson’s diseases: Pathophysiological roles and therapeutic implications. Pharmacol. Res..

[18] Zhang Z.G., Zhang L., Jiang Q., Zhang R., Davies K., Powers C., van Bruggen N., Chopp M. (2000). VEGF enhances angiogenesis and promotes blood-brain barrier leakage in the ischemic brain. J. Clin. Investig..

[19] Balkaya M., Cho S. (2019). Genetics of stroke recovery: BDNF val66met polymorphism in stroke recovery and its interaction with aging. Neurobiol. Dis..

[20] Rroji O., Mucignat C. (2024). Factors influencing brain recovery from stroke via possible epigenetic changes. Future Sci. OA.

[21] Gwoździńska P., Buchbinder B.A., Mayer K., Herold S., Morty R.E., Seeger W., Vadász I. (2017). Hypercapnia Impairs ENaC Cell Surface Stability by Promoting Phosphorylation, Polyubiquitination and Endocytosis of β-ENaC in a Human Alveolar Epithelial Cell Line. Front. Immunol..

[22] Galganska H., Jarmuszkiewicz W., Galganski L. (2021). Carbon dioxide inhibits COVID-19-type proinflammatory responses through extracellular signal-regulated kinases 1 and 2, novel carbon dioxide sensors. Cell. Mol. Life Sci..

[23] Kulikov V.P., Tregub P.P., Kovzelev P.D., Dorokhov E.A., Belousov A.A. (2015). Hypercapnia--alternative hypoxia signal incentives to increase HIF-1α and erythropoietin in the brain. Patol. Fiziol. Eksp. Ter..

[24] Tregub P.P., Malinovskaya N.A., Morgun A.V., Osipova E.D., Kulikov V.P., Kuzovkov D.A., Kovzelev P.D. (2020). Hypercapnia potentiates HIF-1α activation in the brain of rats exposed to intermittent hypoxia. Respir. Physiol. Neurobiol..

[25] Townsend P.D., Holliday P.M., Fenyk S., Hess K.C., Gray M.A., Hodgson D.R., Cann M.J. (2009). Stimulation of mammalian G-protein-responsive adenylyl cyclases by carbon dioxide. J. Biol. Chem..

[26] Zhu G., Liu Y., Zhi Y., Jin Y., Li J., Shi W., Liu Y., Han Y., Yu S., Jiang J. (2019). PKA- and Ca^2+^-dependent p38 MAPK/CREB activation protects against manganese-mediated neuronal apoptosis. Toxicol. Lett..

[27] Haddad J.J., Hanbali L.B. (2014). Hypoxia upregulates MAPK(p38)/MAPK(ERK) phosphorylation in vitro: Neuroimmunological differential time-dependent expression of MAPKs. Protein Pept. Lett..

[28] Sun D., Amiri M., Meng Q., Unnithan R.R., French C. (2024). Calcium Signalling in Neurological Disorders, with Insights from Miniature Fluorescence Microscopy. Cells.

[29] Cook Z.C., Gray M.A., Cann M.J. (2012). Elevated carbon dioxide blunts mammalian cAMP signaling dependent on inositol 1,4,5-triphosphate receptor-mediated Ca^2+^ release. J. Biol. Chem..

[30] Shigemura M., Lecuona E., Angulo M., Homma T., Rodríguez D.A., Gonzalez-Gonzalez F.J., Welch L.C., Amarelle L., Kim S.J., Kaminski N. (2018). Hypercapnia increases airway smooth muscle contractility via caspase-7-mediated miR-133a-RhoA signaling. Sci. Transl. Med..

[31] Nishio K., Suzuki Y., Takeshita K., Aoki T., Kudo H., Sato N., Naoki K., Miyao N., Ishii M., Yamaguchi K. (2001). Effects of hypercapnia and hypocapnia on [Ca2+]i mobilization in human pulmonary artery endothelial cells. J. Appl. Physiol..

[32] Lin C., Pulliam T.L., Han J.J., Xu J., Recio C.V., Wilkenfeld S.R., Shi Y., Kushwaha M., Bench S., Ruiz E. (2025). Cholesterol metabolism regulated by CAMKK2-CREB signaling promotes castration-resistant prostate cancer. Cell Rep..

[33] Li J., Dong S., Quan S., Ding S., Zhou X., Yu Y., Wu Y., Huang W., Shi Q., Li Q. (2024). Nuciferine reduces inflammation induced by cerebral ischemia-reperfusion injury through the PI3K/Akt/NF-κB pathway. Phytomedicine.

[34] Guo Q., Jin Y., Chen X., Ye X., Shen X., Lin M., Zeng C., Zhou T., Zhang J. (2024). NF-κB in biology and targeted therapy: New insights and translational implications. Signal Transduct. Target. Ther..

[35] Keogh C.E., Scholz C.C., Rodriguez J., Selfridge A.C., von Kriegsheim A., Cummins E.P. (2017). Carbon dioxide-dependent regulation of NF-κB family members RelB and p100 gives molecular insight into CO_2_-dependent immune regulation. J. Biol. Chem..

[36] Tang S.E., Wu S.Y., Chu S.J., Tzeng Y.S., Peng C.K., Lan C.C., Perng W.C., Wu C.P., Huang K.L. (2019). Pre-Treatment with Ten-Minute Carbon Dioxide Inhalation Prevents Lipopolysaccharide-Induced Lung Injury in Mice via Down-Regulation of Toll-Like Receptor 4 Expression. Int. J. Mol. Sci..

[37] Lu Z., Casalino-Matsuda S.M., Nair A., Buchbinder A., Budinger G.R.S., Sporn P.H.S., Gates K.L. (2018). A role for heat shock factor 1 in hypercapnia-induced inhibition of inflammatory cytokine expression. FASEB J..

[38] Ding H., Li Y., Li X., Liu X., Chen S., Liu M., Zeng H. (2020). Treatment with 7% and 10% CO_2_ Enhanced Expression of IL-1β, TNF-α, and IL-6 in Hypoxic Cultures of Human Whole Blood. J. Int. Med. Res..

[39] Kimura D., Totapally B.R., Raszynski A., Ramachandran C., Torbati D. (2008). The Effects of CO_2_ on Cytokine Concentrations in Endotoxin-Stimulated Human Whole Blood. Crit. Care Med..

[40] Yang L.-L., Ji X.-P., Liu Z., Liu G., Guan F.-L. (2004). Effects of Hypercapnia on Nuclear Factor-κB and TNF-α in Acute Lung Injury Models. Zhongguo Ying Yong Sheng Li Xue Za Zhi.

[41] Phelan D.E., Mota C., Strowitzki M.J., Shigemura M., Sznajder J.I., Crowe L., Masterson J.C., Hayes S.E., Reddan B., Yin X. (2023). Hypercapnia alters mitochondrial gene expression and acylcarnitine production in monocytes. Immunol. Cell Biol..

[42] Zitta K., Meybohm P., Bein B., Heinrich C., Renner J., Cremer J., Steinfath M., Scholz J., Albrecht M. (2012). Serum from patients undergoing remote ischemic preconditioning protects cultured human intestinal cells from hypoxia-induced damage: Involvement of matrixmetalloproteinase-2 and -9. Mol. Med..

[43] Nadeev A.D., Kritskaya K.A., Fedotova E.I., Berezhnov A.V. (2022). “One Small Step for Mouse”: High CO_2_ Inhalation as a New Therapeutic Strategy for Parkinson’s Disease. Biomedicines.

[44] Chao C.M., Chen C.L., Niu K.C., Lin C.H., Tang L.Y., Lin L.S., Chang C.P. (2020). Hypobaric hypoxia preconditioning protects against hypothalamic neuron apoptosis in heat-exposed rats by reversing hypothalamic overexpression of matrix metalloproteinase-9 and ischemia. Int. J. Med. Sci..

[45] Chen J., Li Y., Wang L., Zhang Z., Lu D., Lu M., Chopp M. (2001). Therapeutic benefit of intravenous administration of bone marrow stromal cells after cerebral ischemia in rats. Stroke.

[46] Modi A.D., Parekh A., Patel Z.H. (2024). Methods for evaluating gait associated dynamic balance and coordination in rodents. Behav. Brain Res..

[47] Balduini W., De Angelis V., Mazzoni E., Cimino M. (2000). Long-lasting behavioral alterations following a hypoxic/ischemic brain injury in neonatal rats. Brain Res..

[48] Keiner S., Wurm F., Kunze A., Witte O.W., Redecker C. (2008). Rehabilitative therapies differentially alter proliferation and survival of glial cell populations in the perilesional zone of cortical infarcts. Glia.

[49] Dell R.B., Holleran S., Ramakrishnan R. (2002). Sample Size Determination. ILAR J..

